# Implementation and preliminary testing of a theory-guided nursing discharge teaching intervention for adult inpatients aged 50 and over with multimorbidity: a pragmatic feasibility study protocol

**DOI:** 10.1186/s40814-021-00812-4

**Published:** 2021-03-17

**Authors:** Joanie Pellet, Marianne Weiss, Franziska Zúñiga, Cedric Mabire

**Affiliations:** 1 Institute of Higher Education and Research in Healthcare, Lausanne University Hospital and University of Lausanne, Lausanne, Switzerland; 2 Marquette University College of Nursing, Milwaukee, WI USA; 3 Nursing Science, Department Public Health, Faculty of Medicine, University of Basel, Basel, Switzerland

**Keywords:** Discharge teaching, Multimorbidity, Hospitalization, Patient activation, Implementation science, Nursing

## Abstract

**Background:**

Discharge teaching by nurses during hospitalization is essential to provide multimorbid inpatients with the knowledge and skills to self-manage their health conditions. However, available disease-specific teaching guidelines do not address the cumulative complexity of multiple chronic diseases that occur with greater frequency in older adults. Therefore, there is a need for a discharge teaching intervention which uses concepts that specifically address the needs of these patients, such as considering their level of activation (i.e. knowledge, skills and confidence to self-manage their health) and the burden of multimorbid disease. The objectives of this pragmatic study will be to (1) test the feasibility of implementing a nursing discharge teaching intervention and (2) conduct a preliminary test of this novel discharge teaching intervention with adult inpatients age 50 or greater who have multiple comorbid conditions.

**Methods:**

This study uses a two-group pre-posttest design. Participants are drawn from medical units in three hospitals in the French-speaking part of Switzerland. The implementation of the intervention will be facilitated by implementation strategies from the Theoretical Domains Framework and the Behavior Change Wheel and will target change in nurses’ teaching behaviours. Implementation outcomes will include measures of feasibility of the implementation strategies and the intervention process. Participants in the intervention group will receive tailored discharge teaching by trained teaching nurses. Patient outcomes will inform the preliminary testing of the intervention and will be measured with validated questionnaires assessing patients’ activation level, health confidence, perceived readiness for discharge, experience with the discharge process and rate of and time to readmission.

**Discussion:**

The study takes a pragmatic approach to examining the feasibility of implementing the discharge teaching intervention to contribute to the knowledge development within the context of the real-world practice setting. Results will provide the foundation for clinical trials to build evidence for widespread adoption of this intervention.

**Trial registration:**

The trial is registered at ClinicalTrials.gov (ID: NCT04253665) on the 30 of January 2020 and has been approved by the Cantonal Ethics Committee Vaud in Switzerland (2020-00141).

**Supplementary Information:**

The online version contains supplementary material available at 10.1186/s40814-021-00812-4.

## Background

Older patients returning home after an acute health event have to manage several chronic diseases within the context of and in addition to their daily tasks. In Switzerland between 2010 and 2011, 79% of hospitalized adults discharged from internal medicine departments were multimorbid [1]. When insufficiently prepared for the transition from hospital care to home, the resulting burden of combined health, mobility, functional and social issues can severely threaten their well-being and lead to costly hospital readmissions [2]. Discharge teaching is a crucial component of discharge preparation as it provides patients with the nec`essary skills and information to self-manage their health at home, i.e. problem-solving skills and abilities to make decisions to improve their health [3–5]. Discharge preparation refers to a multi-faceted care process that aims to prepare patients and their families for their return home. This process consists of three components: discharge planning, discharge coordination and discharge teaching [3]. The latter refers to educational interventions during the hospital stay that aim to provide patients with the knowledge and skills to self-manage their health conditions [3]. High-quality discharge teaching is associated with improved readiness for discharge, adherence to discharge care plans and reductions in mortality, readmission and cost of care [6–10].

To teach multimorbid inpatients, healthcare providers rely mainly on available disease-specific guidelines that do not address this cumulative complexity and resulting treatment burden, nor provide guidance for considering patient preferences [11, 12]. The work of being a patient includes a wide range of activities in terms of understanding diseases, managing treatments, health self-monitoring, visits to doctors, self-care, etc. [13, 14]. To avoid additional burden by intensifying care, prioritizing needs for the return home is one of the keys to multimorbidity management [15, 16]. But while patients usually prioritize the diseases for which they experience the most symptoms or those that interfere with their social activities or independence, health professionals prioritize according to prognosis, severity of the disease and consequences [15]. In planning teaching objectives with patients, it is therefore important to consider the balance between the burden of disease management at home and patient’s capacity to cope with it [15–17]. To ensure that the teaching is appropriate to the patient’s abilities, patient activation also appears to be a crucial characteristic to consider when tailoring the discharge teaching [18]. Activation refers to patient knowledge, skill and confidence for self-management [19]. Deficiencies in current discharge teaching practices for multimorbid inpatients point to the critical need to develop and test a teaching intervention that is not disease-specific and thus applicable to the broad range of patients being discharged from hospital with one or more concurrent conditions.

Several barriers to discharge teaching delivery interfere with effective discharge teaching. In a European study conducted with 33,659 nurses, 41% reported to have left patient education undone on their last shift [20]. Decreasing average lengths of stay, and thereby the time available for discharge preparation, result in limited time to pass on skills, provide targeted information and check whether patients understand their discharge instructions. Units’ workflow often results in patients leaving the hospital on the same day the discharge decision is made, with the consequence that discharge teaching is delivered hurriedly before the patient returns home [21]. In addition to spending too little time on discharge teaching, assessment of patients’ comprehension of discharge instructions is not done in a specific or systematic way [22]. Informational content is forgotten immediately after discharge by 40–80% of patients in acute care, and half of the information received is recalled incorrectly [23]. Patients’ misunderstanding of discharge instructions can lead to noncompliance and ineffective self-management [24]. These issues highlight a discrepancy between professionals’ belief that they address patients’ needs through discharge teaching, the relevance of the content and the adequacy of the way of providing teaching for patients returning home [4, 25, 26].

As discharge teaching practice remains unsatisfactory in French-speaking Switzerland, overcoming implementation barriers requires understanding of the nature of the nurses’ behaviour to be changed and identifying types of intervention that could effectively support these changes [27, 28]. While individual nurses are influenced by organization level policies and practices, we assume that any intervention to improve discharge process would certainly fail without first targeting a practice change in discharge teaching. To facilitate discharge teaching implementation as part of the discharge preparation, the application of a behaviour change framework will support the design of theoretically underpinned strategies that aim to change behaviours at individual nurse level [29–31].

With regard to these different elements, undertaking an effectiveness-implementation hybrid study raises concerns regarding effect size of a non-diseases-specific teaching intervention and necessary implementation strategies to change practices at nurses’ level [29]. We will perform this study to address whether a hybrid type II study will be an appropriate design and feasible with regard to participant recruitment and retention, outcome acceptability, intervention feasibility, acceptability and appropriateness and minimum important difference.

This article outlines a protocol for a feasibility study. The objectives will be to (1) test the feasibility of implementing a novel nursing discharge teaching intervention for older patients (age 50 or more) with multiple chronic conditions hospitalized in medical units and (2) conduct a preliminary test of this novel discharge teaching intervention on multimorbid inpatients’ activation level, health confidence, readiness for hospital discharge, experience with discharge care and rate of and time to readmission.

## Method

### Study design

This study is a pragmatic feasibility study using a two group pre/post-intervention comparative design. This protocol is presented as per the SPIRIT (Standard Protocol Items: Recommendations for Interventional Trials) guideline and Thabane and Lancaster’s guidance on how to report protocols of pilot and feasibility trials [32, 33]. All elements of the SPIRIT checklist adapted with the CONSORT extension are reported in Additional file 1. This study has been approved by the Cantonal Ethics Committee Vaud (2020-00141). Written informed consent will be obtained from eligible patients by the researcher. Nurses participating in the study will receive an information sheet and be asked for written consent.

### Setting

The nursing discharge teaching intervention will be implemented in three medical units, one in a teaching hospital (21 beds, 17 registered nurses) and two in two community-based hospitals (34 beds, 22 registered nurses and 22 beds, 15 registered nurses). The three hospitals are located in the French-speaking part of Switzerland. Medical units were preferred to surgical units because medical patients generally have a higher proportion of older multimorbid patients who have complex discharge needs and higher rates of readmission. Agreement of nurse managers will be the basis for units to participate. They will provide data on contextual and organizational characteristics of their units and will be the contact person for each setting for the coordination of the study process.

### Participants and recruitment

A sample of 12–15 nurses (4–5 per unit) will be recruited on a voluntary basis to take part in the study. Their participation will involve being trained in the intervention, delivering it during the implementation phase, participating in qualitative and quantitative evaluations in the pre- and post-implementation phase of their teaching behaviours and feasibility of the intervention. Inclusion criteria for nurse participants will be registered nurses, employed full time (80–100% work rate).

Inclusion criteria for participants will be 50 years old or more, having two or more chronic conditions, being hospitalized for more than 48 h, an expected discharge home defined at the beginning of the hospitalization and fluent speaking, reading and writing in French [34]. The inclusion criterion of 50 and over was chosen because from this age onwards the prevalence of multimorbidity is constantly increasing [35]. These criteria are deliberately broad in order to obtain a sufficient diversity of patients to reflect the variations encountered in patients in real-world practice. Patients will be excluded if they are cognitively unable to give their informed consent.

As this is a feasibility study, there is no sample size calculation [36]. A convenience sample of 180 hospitalized patients (90 pretest/90 posttest) was determined based on an estimate of the number of patients meeting the criteria for inclusion in the medical units of 30 patients per unit month. With an estimated recruitment rate of 50%, 30 pretest and 30 posttest patients per unit could be recruited over a 2-month period to achieve the 180-patient sample. Pretest participants will be recruited before nurses’ training about discharge teaching to prevent contamination. All eligible patients will be informed about the study and recruited four preselected days per week in two sites and 2 days per week in the third site due to resources’ constraints. Patients in the control and intervention groups will be recruited within 2 days after admission. Patients eligible to participate will be identified by the units’ nurses trained on participants’ inclusion and exclusion criteria. They will routinely screen newly admitted inpatients and inform the researcher of eligible patients. During recruitment days, all eligible patients will be informed about the study and asked for participation.

### Study procedure

Patients’ outcomes will be measured during a control period with patients receiving usual care, followed by outcomes measurements during the implementation period with patients receiving the discharge teaching intervention. The discharge teaching intervention will be implemented as an enhancement to usual care provided to patients hospitalized on the study units and therefore requires change/improvement in nurses’ teaching behaviours to achieve successful outcomes of the teaching intervention. The plan for implementation by clinical nurses of the discharge teaching intervention is based on the Theoretical Domains Framework (TDF) and the Behavior Change Wheel (BCW) of the COM-B model [31, 37].

**Fig. 1:**
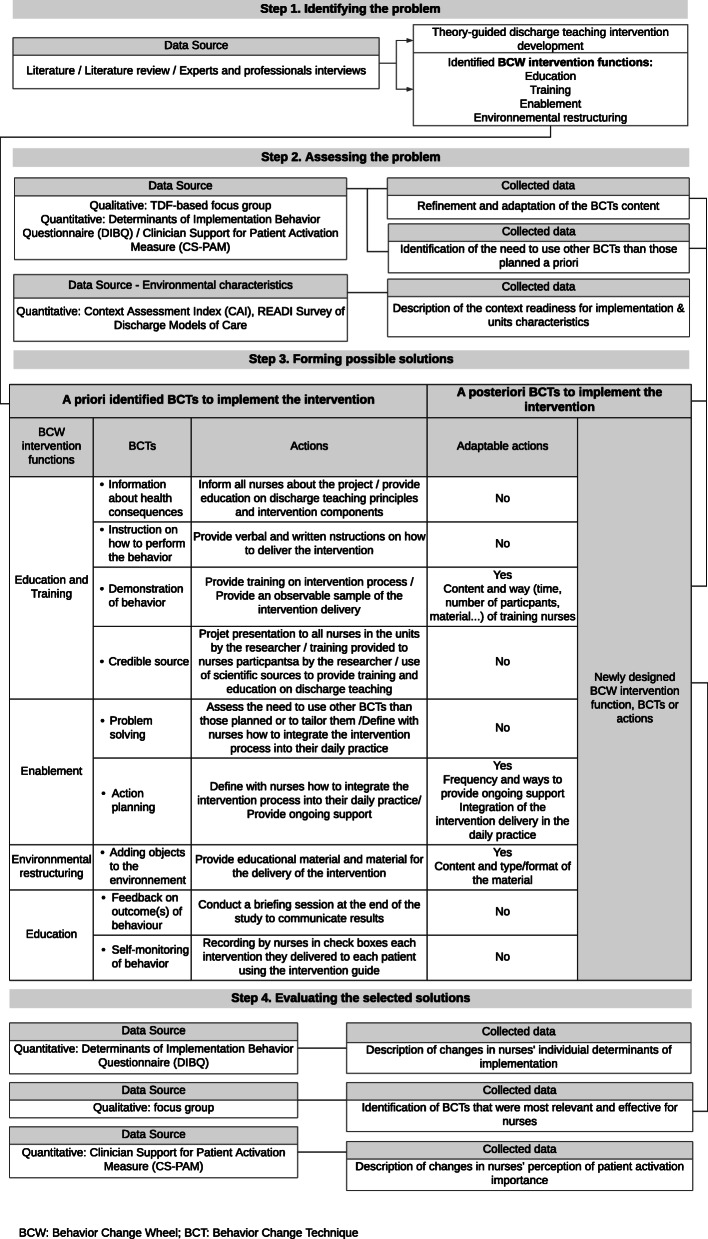
Steps of the implementation plan

The TDF consists in 14 domains identifying influences on health professional behaviour [37]. This framework will be used to identify which domains should be prioritized in implementation approaches to change nurses’ behaviours. These interventions will be informed by the BCW, which is a synthesis of 19 frameworks of behaviour change comprising nine intervention functions [31]. Intervention functions are broad categories of interventions that can change nurses’ behaviours [31]. To develop the implementation plan, we will rely on the four-step method proposed by French et al. [30, 38]. These are identifying the problem, assessing the problem, forming possible solutions and evaluating the selected intervention. Details of steps 1–4 are presented in Fig. 1 and Additional files 2 and 3.

### Intervention

#### Conceptual basis for the intervention

We performed a realist review to shed light on the relational mechanisms triggered between nurses and patients during the interactive discharge teaching–learning process [39]. The realist review resulted in a programme theory of discharge teaching, explaining how the intervention might work and in which circumstances [40]. This programme theory is formed of configurations between context, mechanism and outcomes (CMO configurations) developed from a synthesis of theories of learning and patient education, research literature and expert opinions. Context was defined as the micro-level setting of patient–nurse relationship in which a discharge teaching intervention takes place. Mechanisms are defined as “underlying entities, processes, or structures which operate in particular contexts to generate outcomes of interest” [41]. Outcomes are defined as consequences resulting from intervention activities occurring in a given context [42]. The 10 CMOs identified in the realist review were as follows: (1) information needs, (2) tailored teaching delivery, (3) patient activation, (4) interviewing skills, (5) teaching skills, (6) teachable opportunities, (7) priority concerns about going home, (8) making sense of the hospital stay experience, (9) discharge teaching as a care and (10) involving the caregivers (see Additional file 4) [18]. By generating a more explanatory than descriptive theory of discharge teaching, this programme theory helped us develop an intervention that explains in detail how to tailor the teaching to patients’ needs using the identified mechanisms. To facilitate operationalization of the CMOs, we incorporated four methodological strategies to guide nurses to tailor the teaching to the unique needs of multimorbid older patients: the minimally disruptive medicine model (MDM), patient activation, the patient-oriented discharge summary and the theoretical framework to guide patient/family teaching [14, 43, 44]. Additional files 4 and 5 present how the programme theory, concepts or theories, intervention components, implementation components and outcomes are related.

#### Intervention process

**Fig. 2:**
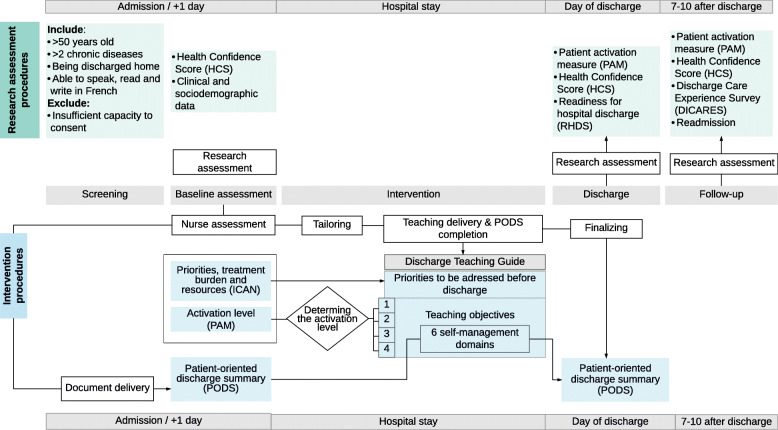
Summary of the discharge teaching intervention process

The discharge teaching intervention begins by identifying *priorities to address related to patients’ life situation* (using the Instrument for Patient Capacity Assessment) and determining the patient’s current *level of activation* (using the Patient Activation Measure) [43, 45]. A Discharge Teaching Guide developed by the Principal Investigator helps to *individualize the teaching* to the patient’s priorities and level of activation. It will be used by trained teaching nurses to deliver the teaching and includes six domains of self-management. These *six domains of self-management* correspond to those of the patient-oriented discharge summary (PODS), which is a document provided to the patient by the teaching nurse that summarizes what has been addressed in discharge teaching. For each domain, teaching nurses first report in the Discharge Teaching Guide whether a priority has been identified with the Instrument for Patient Capacity Assessment (ICAN) and what intervention they have proposed to address it. Then for each domain, this guide provides teaching objectives which differ according to the level of patient activation previously evaluated with the Patient Activation Measure (PAM) (Fig. 2).

#### Assessment measures used by nurses in the intervention

##### Patients’ life situation

The first element of the teaching intervention is assessment of the patients’ life situation, assessed with the ICAN. The ICAN was developed from a systematic review on patients’ capacity and a user-centred design of a discussion aid about patient capacity [45]. Observations during clinical encounters and nurse home visits were followed by the development and testing prototypes, resulting in the final ICAN Discussion Aid (see Additional file 6). The ICAN was found feasible to use during clinical encounters [46]. Using videographic coding scheme, results also showed that issues discussed with the ICAN were seldom discussed during control clinical encounters [46]. A randomized controlled trial is ongoing to test the impact on patient and healthcare teams’ care experience and communication, while reducing patient treatment burden (NCT03017196).

Teaching nurses will give the ICAN to participants in a leaflet form as soon as they are enrolled early in the hospitalization, explaining that it will help them to learn more about their lives and how that interacts with their health and healthcare. Participants will be asked to check relevant boxes related to 11 life domains and eight clinical demands to classify these as source of burden or satisfaction/help [45]. The ICAN will remain on the patient’s bedside table so that the nurse who will deliver the teaching can read it beforehand. Nurses will check with participants if there are any priorities among what they identified as a burden in the different domains that should be addressed for their return home. Clinical demand domains listed in the ICAN are linked to the self-management domains of the Discharge Teaching Guide. Before starting to teach one of the areas of self-management, teaching nurses must first check with patients whether there is a priority in terms of clinical demand to be addressed and, if so, will be instructed to briefly describe the proposed intervention to address it. Attending to relevant patient priorities and burdens promote patients’ workload-capacity balance and prioritization of care [47]. The ICAN will also be useful in identifying possible resources that patients could mobilize.

##### Patient activation

Participants will be asked by teaching nurses to complete the Patient Activation Measure (PAM). The PAM is a 13-item self-report questionnaire to measure patient activation level [43]. Patients can go through four levels of activation: (1) disengaged and overwhelmed, (2) becoming aware but still struggling, (3) taking action and (4) maintaining behaviours and pushing further. Stages of activation are distributed as follows in the PAM items: items 1–2, believing an active role is important; items 3–8, having confidence and knowledge to take action; items 9–11, taking action; and items 12–13, continuing healthy behaviours under stress. Responses range from 1 = “strongly disagree” to 4 = “strongly agree”. PAM raw score can be calculated by adding all of the responses to the 13 questions. This score is then converted into an activation score ranging from 0 = no activation to 100 = high activation using a scoresheet provided by Insignia Health®. Psychometric properties of the PAM in hospitalized multimorbid patients showed a satisfying reliability (Cronbach’s alpha = 0.88) and a content validity index of 0.91 [48]. Another study in the inpatient setting provided evidence for the internal consistency reliability (Cronbach’s alpha = 0.81) and the construct validity of the PAM-13 [49]. A PAM research license will be obtained from Insignia Health®, which will provide a validated French version of the PAM-13 and the scoresheet [50]. The level of activation calculated with the PAM will define which category of Discharge Teaching Guide the nurses will use. The guide corresponding to the participant activation level will guide them towards which objectives related to self-management domains are realistic based on this level. Levels 1 and 2 describe patients who are rather passive recipients of care and who lack basic health knowledge, for whom the objectives aim at increasing the awareness or basic knowledge of their health information. Teaching objectives for patients with a moderate level of activation (level 3) aim to support them in developing their knowledge and skills in managing their health and encourage them to play a more active role in their care. For patients with a high level of activation (level 4), the objectives aim to complete missing knowledge and help them to prevent relapses.

#### Teaching methods used by nurses to deliver the intervention

##### Discharge Teaching Guide

The Discharge Teaching Guide was designed to guide nurses in how to conduct discharge teaching incorporating patient priorities identified using the ICAN, patient activation level identified using the PAM, and the six self-management domains identified in the PODS: reason for hospitalization, warning signs, medication plan, health behaviours, next appointments and which person to contact if needed. In the Discharge Teaching Guide, a first row for each domain of self-management reminds nurses to first check with the ICAN if patients experience any burden or priority that should be addressed for their return home. If this is the case, nurses must then report in the guide the proposed intervention to address this burden or priority. The Discharge Teaching Guide then supports nurses in addressing teaching objectives in each domain of self-management tailored to the patient's level of activation and suggests interventions to achieve these objectives. The guide also includes checkboxes to remind nurses to complete the PODS (see below) with patients to summarize the teaching content discussed for the management of their health at home. Teach-back is also recalled as a technique to be used to promote patient understanding and will have been taught to nurses during the intervention training.

##### Patient-Oriented Discharge Summary

Patient-Oriented Discharge Summary (PODS) is a simple tool, containing meaningful information for patients presented in an easy-to-understand format [44]. This summary is a one-page document, with key information to be completed, such as the reason for hospitalization, warning signs to be monitored, who to call depending on the problem, the treatment plan and upcoming medical appointments. Participants will receive it at the beginning of the intervention, at the same time as the ICAN and the PAM. The PODS will be filled out during the hospital stay by the participant and the teaching nurse as teaching relevant to discharge occurs. Teaching nurses will review and verify on the day of discharge that the information written in the PODS correspond to the teaching content that was discussed (Fig. 2). Participants take the PODS home at discharge to serve as a reference for relevant and individualized discharge information in the post-discharge period.

### Data collection

**Fig. 3:**
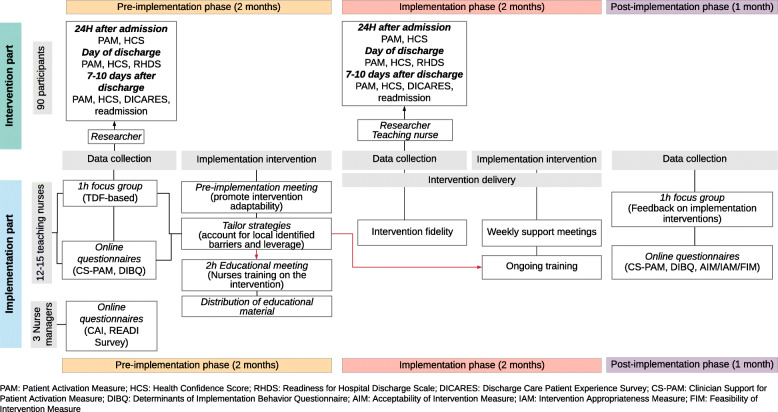
Data collection and implementation process

Study data will be collected and managed using REDCap™ electronic data capture tools hosted at the Lausanne University Hospital. Collection of both quantitative and qualitative data for the objective 1 will comprise (a) online questionnaires completed by the teaching nurses at pre- and post-implementation phases to assess changes in barriers to implementation and beliefs and attitudes regarding the importance of patient activation; (b) online questionnaires at pre-implementation phase for nurse managers to assess units’ readiness for research utilization in practice and structural and organizational environment; (c) a TDF-based focus group with teaching nurses at pre-implementation phase to qualitatively assess perceived barriers to discharge teaching; (d) online questionnaires at post-implementation phase for teaching nurses to assess feasibility, acceptability and appropriateness of the intervention; (e) self-reported measure by teaching nurses of intervention fidelity during implementation phase; and (f) a focus group with teaching nurses at post-implementation phase to assess the appropriateness and the relevancy of the implementation strategies (Fig. 3).

Collection of quantitative data for the objective 2 will comprise (a) patient participants questionnaires at the beginning of the hospital stay, the day of discharge and a telephone survey 7–10 days after discharge and (b) chart reviews to collect participants’ socio-demographic and medical data, and readmissions within 7 days (Figs. 2 and 3).

### Study measures

#### Objective 1: Test the feasibility of implementing the nursing discharge teaching intervention

*Changes in behavioural determinants* regarding the implementation of the intervention will be assessed with the Determinants of Implementation Behavior Questionnaire (DIBQ), which is based on the TDF domains [51]. The DIBQ will be completed by teaching nurses at pre- and post-implementation phase. The questionnaire comprises 93 items, investigating 18 domains of behavioural determinants: knowledge, skills, social/professional and role and identity, beliefs about capabilities, optimism, beliefs about consequences, intentions, goals, innovation, socio-political context, organization, patient, innovation strategy, social influences, positive emotions, negative emotions, behavioural regulation and nature of the behaviours. Responses are scored from 1 (strongly disagree) to 7 (strongly agree). Discriminant content validity with the TDF domains resulted in items discriminately assessing 11 out of the 14 domains [52]. Internal consistency values of the 18 domains range from 0.68 to 0.93 [51]. The DIBQ will be translated into French according to Wild’s method [53].

*Changes in nurses’ attitudes* regarding the importance of patient self-management behaviours will be evaluated with the Clinician Support for Patient Activation Measure (CS-PAM) [54]. The CS-PAM will be completed by teaching nurses at pre- and post-implementation phases. The CS-PAM consists of 14 items taken from the PAM and prefaced with the question “As a clinician, how important is it to you that patients…”. Responses are scored on a 4-point Likert scale from *Extremely important* to *Not important*. Data of CS-PAM will be sent to Insignia Health®, the licensing rights supplier for this instrument, who will score the data and send the results back [50]. This 14-item measure had a Cronbach’s alpha of 0.86, and Rasch analysis provided an overall person reliability of 0.80 tested with primary care clinician [54]. As no French-version is available, the CS-PAM will be translated according to Wild’s method [53].

*Acceptability, appropriateness and feasibility* of the intervention will be evaluated in the post-implementation phase by teaching nurses with three implementation outcome measures recently developed by Weiner et al. [55] to assess the success of implementation efforts: Acceptability of Intervention Measure (AIM), Intervention Appropriateness Measure (IAM) and Feasibility of Intervention Measure (FIM). Each measure has 4 items, with scale values ranging from 1 (completely disagree) to 5 (completely agree). Higher scores indicate greater acceptability, appropriateness and feasibility. These three measures have demonstrated promising psychometric properties: Cronbach alphas of 0.85 for AIM, 0.91 for IAM and 0.89 for FIM and test-retest reliability of 0.83 (AIM), 0.87 (IAM) and 0.88 (FIM) [55].

*Fidelity* of the discharge teaching delivery will be determined by entry of intervention activities on the Discharge Teaching Guide. Nurses will use a Discharge Teaching Guide for each patient enrolled in the study, on which they will record in check boxes each intervention they delivered to their patient. A descriptive interpretation will be done to analyse the level of fidelity.

*Appropriateness* of implementation strategies will also be explored through a focus group conducted by the researcher with teaching nurses during the post-implementation phase. The nurses will be asked which implementation strategies were most useful to them in facilitating the implementation and the delivery of the discharge teaching intervention.

#### Objective 2: To conduct a preliminary test of the nursing discharge teaching intervention

##### Health confidence

The Health Confidence Score (HCS) is an easy-to-obtain proxy measure for the patient activation construct [56]. As this instrument has been very recently validated, there are currently no other validation studies or studies that have tested that it as an activation proxy. However, many authors in the field agree on this fact, knowing that confidence is one of the underlying concepts of patient activation [57, 58]. The HCS is a short measure (4 items) of patients’ confidence to manage their health and engage with healthcare providers [56]. Four dimensions are explored: knowledge, self-management, access to help and shared decision-making. Items have four response options (3=strongly agree, 2=agree, 1=neutral, 0=disagree). Scores are reported for each item and the higher the score, the higher the confidence. A summary score is calculated with a 13-point scale by adding the individual items scores, with a range from 0 (4×disagree) to the ceiling 12 (4×strongly agree). This instrument has good internal consistency (Cronbach’s alpha = 0.82) and construct validity [56]. The HCS will be translated in French according to Wild’s method [53].

##### Readiness for hospital discharge

The Readiness for Hospital Discharge Scale–Short Form (RHDS-SF) is an eight-item self-reported questionnaire [59]. Four dimensions measure personal status, knowledge to manage the post-hospital period, the ability to adapt to new health needs and the expected support [59]. Each item is scored on a Likert scale from 0 to 10, with the highest score indicating better perceived readiness. A mean score of less than 7 indicates low ready for hospital discharge [6, 60]. The short form of the RHDS explains 93% of the scale variance and reliability was 0.79 [59]. Results of predictive validity for older people showed that patients with higher scores were less likely to be readmitted (OR = 0.89, CI (95%) = 0.80-0.98, *P* = 0.03) [61]. The nine-item RHDS-SF for older people available in French will be used although the validation has not yet been carried out [61].

##### Rate of and time to readmission

Rate of and time to readmission will be measured by consulting administrative hospitalization data 7 days after patient discharge. Only readmission for the index hospitalization will be included.

##### Experience with discharge care

Patients’ experience with discharge care process will be assessed 7 to 10 days after discharge with the 11 items of the Discharge Care Experiences Survey (DICARES) [62]. This instrument investigates three domains: coping after discharge (4 items), adherence to treatment (3 items) and participation in discharge planning (4 items). The answer for each item ranges from 1 (“Not at all”) to 5 (“To a very large extent”), with higher scores indicating more positive experience. The psychometric evaluation of the DICARES in older patients showed an excellent test-retest reliability (ICC=0.76, CI 95; 0.70, 0.82), satisfactory construct validity (rho = 0.54, *p* <0.01) and an acceptable internal consistency (Cronbach’s alpha = 0.82) [62, 63].

##### Sociodemographic and medical characteristics

The following baseline participants sociodemographic and health data will be collected from health electronic records and reported in a REDCap™ electronic case report form (eCRF): age, sex, number of chronic conditions, years living with a chronic disease, length of stay and use of home health care services before and after index hospitalization. To determine the number of chronic conditions, a list of 75 chronic conditions most relevant in multimorbidity and developed by experts in Switzerland will be used to retrieve data on chronic conditions from discharge data from electronic records and coded as 1=the condition is present and 0= the condition is absent [64]. Total number of chronic conditions will be calculated for each patient. Number of previous hospitalizations in the same hospital within the last year will be retrieved from administrative hospitalization data and patients will be asked for information about prior hospitalization in a different hospital. As cohabitation and education are usually not specified in health electronic records, these questions will be asked directly to the participants.

### Data analysis

#### Objective 1: Test the feasibility of implementing the nursing discharge teaching intervention

Domains-level data of the Determinants of Implementation Behavior Questionnaire (DIBQ) will be analysed as categorical data. The proportion of nurses responding “agree” or “strongly agree”, indicating that the domain is a facilitating determinant of implementation behaviour, will be reported for items and domain. The same will be done for hindering domains indicated by “disagree” or “strongly disagree” responses.

CS-PAM responses will be reported in a scoring spreadsheet provided by Insignia Health® and will be sent to the license supplier who will calculate and send back the scoring. Assessment of differences in nurses’ beliefs and attitudes about patient activation between pre- and post-implementation phase will be undertaken through comparison tests (Student’s *t*-test).

Descriptive statistical analyses will be conducted to obtain means and standard deviations of Acceptability of Intervention Measure (AIM), Intervention Appropriateness Measure (IAM) and Feasibility of Intervention Measure (FIM). Higher scores will indicate greater acceptability, appropriateness and feasibility. The intervention will also be considered as feasible within clinical practice and for pragmatic clinical research if the recruitment rate is higher than 50%, the loss to follow up is less than 20% and more than 80% of outcome measure are completed. Progression criteria that will inform the decision to conduct a larger and definitive trial are detailed in Additional material 7 [65].

Content analysis of focus groups will also be carried out to provide information on behavioural determinants for the intervention, as well as acceptability, appropriateness and feasibility of the implementation strategies [66]. The focus groups will be transcribed in verbatim and read through several times first to get a sense of the whole. Transcripts will be coded deductively, using the 18 TDF domains as well as acceptability and feasibility of the implementation strategies as categories. The results will be discussed in the research group on the one hand linking them to the results of the DIBQ survey and to understand more fully behavioural determinants for implementation, and on the other hand to explore, based on the implementation outcomes, which implementation strategies could be used best in future trials.

#### Objective 2: Conduct a preliminary test of the nursing discharge teaching intervention

Descriptive statistical analyses of all variables will be conducted to obtain means and standard deviations for continuous variables and proportions for categorical variables. Assessment of differences in patient activation, health confidence, readiness for hospital discharge, experience with discharge care and readmission rates between pre and post-test participants will be undertaken through comparison tests (Student’s *t* test and chi-square test). Effect size and confidence interval of the intervention will be estimated to be able to calculate the sample size for future efficacy studies [67]. Effect size of the intervention will be estimated with a linear regression model of level of patient activation and health confidence at discharge on levels measured at admission. The adjusted model will include following variables: age, sex, level of education and number of chronic diseases. In light of our sample size, effect size will be interpreted with consideration of both the magnitude and *p* value [68]. All analyses will be carried out using Stata 16 computing software [69].

## Discussion

This paper presents a study protocol for judging the feasibility of a nursing discharge teaching intervention for inpatients with chronic conditions in French-speaking Swiss hospitals. Adopting a pragmatic approach, the planned feasibility study has the potential to generate a better understanding of how an intervention can be implemented and tested in “real-world” clinical practice [70]. Most of nursing activities are guided by descriptive frameworks of interventions, prescribing what nurses should do. The discharge teaching intervention proposed in this study differs precisely because it has been developed on the basis of an explanatory model of how discharge teaching works and under what circumstances [39].

The proposed innovative discharge teaching intervention uses the patient activation concept and patients’ life situation to individualize and better tailor the teaching. Taking these factors into account is expected to contribute to changes in how nurses prepare patients for discharge. In addition, this study will also generate new knowledge in the patient teaching field because the activation concept has not yet been applied in the context of preparation for discharge from an acute hospitalization. Results will therefore contribute to determining whether older and multimorbid inpatients could benefit from applying the concept of patient activation to discharge teaching.

As implementation of discharge teaching remains unsatisfactory in French-speaking Switzerland, a preliminary step appears necessary to enhance nurses’ awareness about the importance of discharge teaching [27]. Any intervention to improve discharge process would certainly fail without targeting a practice change in discharge teaching. The use of an implementation framework will help us to generate an understanding of factors at nurses’ individual level that contribute to the success or failure of the intervention implementation. Thus, understanding how to successfully implement discharge teaching in the real-world practice will help to remove obstacles often encountered when implementing interventions initially tested in highly controlled conditions, in the real-world practice. Results will inform future design of a hybrid type II trial to determine effectiveness of the discharge teaching intervention and implementation approaches [29].

## Supplementary Information

**Additional file 1.** SPIRIT checklist. All elements of the SPIRIT checklist

**Additional file 2.** Implementation strategies. Detailed information about implementation strategies and related actions, their dose and temporality, and related implementation measures.

**Additional file 3.** Steps of the implementation part. Detailed process for the implementation strategies development according to the four-steps method proposed by French et al. (2012).

**Additional file 4.** Theory-guided intervention of nursing discharge teaching. Link between the programme theory resulting from the realist review, related concepts and theories, intervention components, implementation components and outcomes.

**Additional file 5.** Conceptual basis for the intervention. Link between the programme theory resulting from the realist review, related concepts and theories and intervention components.

**Additional file 6.** ICAN domains. Life and clinical domains of the ICAN tool. Patients have to classify these as source of burden or satisfaction/help.

**Additional file 7.** «Stop » and « Go » progression criteria. Progression criteria that will inform the decision to conduct a larger and definitive trial.

## Data Availability

Not applicable

## Abbreviations

AIM: Acceptability of Intervention Measure
BCW: Behavior Change Wheel
CMO: Context-Mechanism-Outcomes
CS-PAM: Clinician Support for Patient Activation Measure
DIBQ: The Determinants of Implementation Behavior Questionnaire
DICARES: Discharge Care Experiences Survey
eCRF: Electronic case report form
FIM: Feasibility of Intervention Measure
HCS: Health Confidence Score
IAM: Intervention Appropriateness Measure
ICAN: Instrument for Patient Capacity Assessment
MDM: Minimally disruptive medicine
PAM: Patient Activation Measure
PODS: Patient-Oriented Discharge Summary
RHDS-SF: Readiness for Hospital Discharge Scale–Short Form
TDF: Theoretical Domains Framework
UBACC: University of California San Diego Brief Assessment of Capacity to Consent
