# Supplementary material for: Implementation and preliminary testing of a theory-guided nursing discharge teaching intervention for adult inpatients aged 50 and over with multimorbidity: a pragmatic feasibility study protocol
Source: Pilot Feasibility Stud. 2021 Mar 17;7:71. doi: 10.1186/s40814-021-00812-4 (PMC7968193; doi:10.1186/s40814-021-00812-4)
Supplement: Supplementary file 2 — Additional file 2. Implementation strategies. Detailed information about implementation strategies and related actions, their dose and temporality, and related implementation measures. [file 40814_2021_812_MOESM2_ESM.docx]

Additional file 2. Implementation strategies

| **BWC Intervention functions** | Education  Training | Enablement | Environnemental restructuring |
| --- | --- | --- | --- |
| **Individual BCTs** | Information about health consequences  Instruction on how to perform the behavior  Demonstration of behavior  Credible source  Self-monitoring of behavior  Feedback on outcome(s) of behaviour | Problem solving  Action planning | Adding objects to the environnement |
| TDF domains | Knowledge, Skills, Beliefs about capabilities, Behavioral regulation, Reinforcement | Goals, Environmental context and resources | Environmental context and resources |
| Actors | Investigator | Investigator | Investigator |
| Participants | Nurse managers, unit nurses, clinician nurse specialists | Recruited nurses, nurse managers | Recruited nurses, nurse managers/clinician nurse specialists |
| Actions | **Pre-implementation phase:**   - Inform all nurses about the project and invite them to participate - Provide recruited nurses training on discharge teaching principles and intervention components - Provide educational material (posters describing the intervention process, Discharge Teaching Guide in pocket cards format and A4 paper sheets)   **Implementation phase:**   - Give nurses a training refresher on the intervention during implementation phase - Ask nurses to self-report teaching interventions delivered to teach patients in the Discharge teaching Guide   **Post-implementation phase**:   - Conduct post-implementation meetings to communicate the results of the study | **Pre-implementation phase:**   - Conduct pre-implementation focus group with nurses to assess the need to use other BCTs than those planned or to tailor them - Tailor intervention training according to identified barriers - Conduct pre-implementation meeting to define with nurses how to integrate the intervention process into their daily practice (e.g., where to place the documents needed to deliver the intervention, how to communicate between nurses what elements of teaching have already been delivered, how to ensure that patients will complete the documents forming part of the intervention) - Discuss with nurses once the intervention is initiated whether there are any changes that should be made in the intervention process to ensure that it is delivered as well as possible   **Implementation phase:**   - Provide ongoing support (weekly meetings and availability by phone) | **Pre-implementation phase:**   - Ask the three nurse managers or clinician nurse specialists of the units to revise and suggest adaptation of the Discharge Teaching Guide content and layout - Provide educational material in the units (posters describing the intervention process, Discharge Teaching Guide in pocket cards format and A4 paper sheets) |
| Targets | Interest, engagement, build ownership of the intervention, prepare units for implementation  Build knowledge, skills, confidence  Information and access to intervention resources, | Awareness of settings’ perceived barriers and facilitators and attitudes  Development of tailored implementation approaches  Assess post-implementation changes in barriers/facilitators and attitudes  Compatibility of the intervention with the current discharge process | Compatibility of the intervention with the current discharge process |
| Temporality  Dose | 2h training in pre-implementation  1x 45min refresh meeting over the course of the intervention implementation  Educational material: During implementation, for continuous use  1x45min meeting to communicate results | 1h- pre-implementation focus group  1h-pre-implementation meeting  Throughout the implementation | 1h meeting with nurse managers or clinical nurse specialist to discuss suitability of the Discharge Teaching Guide for daily practice |
| Implementation outcomes addressed | Improved organizational capacity to implement  Nurses attitudes regarding patient activation  Fidelity | Barriers, facilitators, acceptability, feasibility, nurses’ attitudes towards importance of patients’ self-management behaviors | Appropriateness, acceptability |
| Implementation measures | The Clinician Support for Patient Activation Measure (CS-PAM) | The Determinants of Implementation Behavior Questionnaire (DIBQ), The Clinician Support for Patient Activation Measure (CS-PAM), Acceptability of Intervention Measure (AIM), Intervention Appropriateness Measure (IAM), and Feasibility of Intervention Measure (FIM) | Acceptability of Intervention Measure (AIM), Intervention Appropriateness Measure (IAM) |

(Adapted from Michie, S., Atkins, L., & West, R. (2014). The behaviour change wheel : a guide to designing interventions. (37))
