# Supplementary material for: Implementation and preliminary testing of a theory-guided nursing discharge teaching intervention for adult inpatients aged 50 and over with multimorbidity: a pragmatic feasibility study protocol
Source: Pilot Feasibility Stud. 2021 Mar 17;7:71. doi: 10.1186/s40814-021-00812-4 (PMC7968193; doi:10.1186/s40814-021-00812-4)
Supplement: Supplementary file 3 — Additional file 3. Steps of the implementation part. Detailed process for the implementation strategies development according to the four-steps method proposed by French et al. (2012). [file 40814_2021_812_MOESM3_ESM.docx]

Additional file 3. Steps of the implementation part

**Step 1: Identifying the problem: who needs to do what, differently?** Discharge teaching is part of the routine of nurses' clinical practice for hospital discharge preparation and has strong supporting evidence (77). Prior to the present study, the problem was identified from the literature and from our previous studies; discharge teaching is not consistently done as part of the discharge preparation in French-speaking Switzerland, teaching is often conducted but informally, even unconsciously, and teaching is usually not based on a practical intervention model and rarely documented by nurses (33, 44, 45). Barriers to effective discharge teaching also already emerged from our literature review and interviews with experts and professionals: nurses' lack of knowledge and skills on patient teaching, little time available, the priority interest of new nurses in technical care, and the fact that teaching is often given in a condensed manner just before discharge. The existing body of literature and our literature review helped us to develop an intervention that facilitates discharge teaching delivery for nurses and also identifies a priori intervention functions proposed in the BCW likely to be effective to address identified barriers (Step 3). As a result, the targeted behaviour for nurses will be to deliver discharge teaching following the proposed intervention protocol.

**Step 2: Assessing the problem: using a theoretical framework, which barriers and enablers need to be addressed?** To gain a more in-depth understanding of local and individual barriers to discharge teaching delivery, we will use the TDF to identify nurses’ determinants of change. Quantitative data will be collected with *The Determinants of Implementation Behavior Questionnaire - DIBQ* derived from this framework (58). Qualitative data will be collected in a focus group based on the TDF conducted by the researcher with teaching nurses. An interview guide based on the Theoretical Determinant Framework will be used to explore the following domains regarding discharge teaching delivery: knowledge, skills, memory/attention/decision process, social/professional role and identity, beliefs about capabilities, motivation and goals, beliefs about consequences, and environmental context and resources. Data analysis of the TDF-based focus group will follow the Framework Method outlined by Gale et al. and will be carried out using MAXQDA Analytics Pro (78, 79). Using a deductive approach, pre-defined codes will be determined from the 14 Theoretical Domains Framework (TDF) for the focus group transcript. Two research team members will independently analyse the transcripts by coding phrases to the TDF pre-defined codes. They will compare the codes they have applied and come to consensus. The frequency of TDF domain coded phrases will be calculated to identify the most often reported TDF domains and thus those that can be targeted as levers of change.

Analysis of data from both the focus group and questionnaire will help us to identify TDF domains that are most likely to influence the implementation of the discharge teaching intervention and associated behaviours (Figure 2). This “behavioural diagnosis” will then guide us to design and adapt behaviour change interventions to improve the implementation (43) (Step 3).

As work environment has also an influence on nurses individual behaviour, we will collect data regarding setting context as a baseline description of units’ characteristics. We will ask nurse managers to assess units’ context using the *Context Assessment Index* (CAI) (80). The CAI has been developed to assess context readiness for research utilisation in practice (80). Based on the Promoting Action on Research Implementation in Health Services Framework (PARIHS), the CAI comprises 37 items, investigating three elements of the context: culture, leadership and evaluation (81). The final score plotted along the continuum from weak to strong context indicates the receptiveness for change. The CAI will be translated into French according to Wild’s method (60). To describe structural and organizational characteristics of the units related to discharge preparation, nurse managers will also complete *the READI Survey of Discharge Models of Care* (82). This survey includes closed-ended questions related to units characteristics (i.e.number of beds, main diagnoses) and the discharge process (i.e. discharge teaching, performed activities, discharge roles).

**Step 3: Forming possible solutions: which intervention components could overcome the modifiable barriers and enhance the enablers?**

Barriers identified from the literature and experts’ interviews (Step 1) were matched with a range of possible BCW intervention functions likely to change behaviours related to those barriers. These intervention functions are: education, training, enablement and environmental restructuring (37). Then, we identified from the taxonomy developed by Michie et al., behaviour change techniques (BCTs) which could deliver the identified intervention function (37, 83). BCTs are active components of an intervention that aim to change a behaviour (37). We also considered other criteria, such as to what extent BCTs were affordable, practical or acceptable in the context of the present study (37). For each BCTs, specific actions are planned and will be carried out as part of the implementation process of this study.

Some of the planned BCTs and corresponding actions may be adapted or modified during the study process, according to the data collected in step 2 with TDF-based focus group and the DIBQ questionnaire (84). Individual barriers not yet considered in step 1 could arise and require adapting some actions or planning new ones. As shown in Figure 2, some a priori planned actions could be considered for modification. For example, content of nurses’ training on the intervention and the way it will be delivered could be adapted if nurses point out during TDF-based focus group knowledge, skills or confidence issues not previously identified. Modalities of ongoing support during the implementation phase and merging the intervention process in units daily practice will mainly be defined by barriers and faciliators identified by nurses during a pre-implementation meeting. Analysis of the DIBQ will also indicate if some TDF-domains influencing nurses’ implementation behaviour were not identified in Step 1 and would require additional BCTs and actions.

**Step 4: Evaluating the selected solutions: how can behaviour change be measured and understood?**

Step 4 will address the second objective of the study, which is to assess whether the implementation approaches selected in step 3 and conducted during the study process will change nurses’ attitudes regarding patient activation, and perceived barriers and facilitators regarding discharge teaching delivery. At the end of the intervention implementation period, we will ask teaching nurses to complete the DIBQ used in step 1 again (58). This will allow us to compare whether or not the identified barriers and facilitators to the teaching delivery have changed after delivering the intervention and participating in the implementation process. At the post-implementation phase, we will also conduct a focus group to gain a more in-depth understanding of which implementation approaches were most useful to nurses in facilitating the implementation of the intervention. This will provide important information on which interventions are likely to be appropriate and effective in future studies and implementation initiatives. Finally, as patient activation is the core component of the intervention, we will also assess changes in nurses perceptions regarding this concept before and after having delivered the intervention during the implementation phase using the *Clinician Support for Patient Activation Measure (CS-PAM) (61)*. This will allow us to examine if providing education and training to the teaching nurses as implementation approaches might influence on perceptions regarding patient activation and ultimately their investment in the concept as an important component of the discharge teaching process.
