# Supplementary material for: Implementation and preliminary testing of a theory-guided nursing discharge teaching intervention for adult inpatients aged 50 and over with multimorbidity: a pragmatic feasibility study protocol
Source: Pilot Feasibility Stud. 2021 Mar 17;7:71. doi: 10.1186/s40814-021-00812-4 (PMC7968193; doi:10.1186/s40814-021-00812-4)
Supplement: Supplementary file 4 — Additional file 4. Theory-guided intervention of nursing discharge teaching. Link between the programme theory resulting from the realist review, related concepts and theories, intervention components, implementation components and outcomes. [file 40814_2021_812_MOESM4_ESM.docx]

Additional file 4. Theory-guided intervention of nursing discharge teaching

| Programme theory  CMO |  | *CMO7. Priority concerns about going home*  *If patients are sufficiently reassured that they will return home after hospital, then they are more able to project themselves into the post-hospital period and nurses can help them to identify and respond to what they are most concerned about for their return home. When patients feel that their needs for the returning home are being taken into account by nurses, a partnering relationship is established, and interventions are consistent with patient priorities.* | *CMO3. Patient activation*  *If nurses assess patients’ activation level, this knowledge of the activation level will help them to target appropriate teaching objectives to offer patients the opportunity to experience small successes that will increase confidence and self-efficacy. Teaching objectives targeting realistic behaviour can increase activation level and improve self-management behaviours.*  *CMO1. Information needs*  *If the discharge teaching content is delivered according to patient's perceived information needs, then patients will perceive this content as relevant for them and will remember it better. When patients remember information that is relevant for them, they will feel more ready to go home and post-discharge unmet needs could be decrease.*  *CMO2. Tailored teaching delivery*  *If the teaching strategy and materials are tailored to patients’ learning style and health literacy, the information will be more meaningful and understandable for patients, leading to better recall of discharge instructions.* | *CMO8. Making sense of the hospital stay experience*  *If patients participate in completing a discharge summary document that is patient-centered and personalized, then its content summarizing relevant information will help patients make sense of their critical illness experience. Making sense of their experience has the potential to increase patient engagement in care and returning home with a discharge summary is helping them to better recall instructions*. | *CMO5. Teaching skills*  *If teaching sessions are conducted by nurses with high-quality teaching skills, then an appropriate amount and content of teaching will be shared, fostering patient’s retention of information. Appropriate content can improve patient’s feeling of being ready for discharge and lead to better post-discharge coping.*  *CMO 4. Interviewing skills*  *If nurses use a motivational interviewing style to discuss self-management behaviour, then this will help patients to explore their barriers and own resources to make small changes for their health, leading to increased confidence to change and skills for effective self-management.* | *CMO9. Discharge teaching as a care*  *If discharge teaching is considered as care in itself, then scheduled formal teaching-learning session will foster the attentiveness of the patient, leading to engagement in the learning process.*  *CMO10. Involving the caregivers*  *If the healthcare team recognizes the role of caregivers, then they will encourage their presence during discharge teaching, which will help patients to decode instructions and ask for clarification if necessary. The involvement of caregivers in discharge teaching sessions leads to patients better understanding and adherence to discharge instructions.* |
| --- | --- | --- | --- | --- | --- | --- |
| Theoretical Underpinnings |  | Prioritization – Minimally Disruptive Medicine Model (May, 2009) (50) | Patient activation (Hibbard, 2014) (65) | Patient-oriented discharge summary (Hahn-Goldberg, 2016) (51) | Theoretical framework to guide patient/family teaching (Candela, 2018) (31) | Theoretical framework to guide patient/family teaching (Candela, 2018) (31) |
| Component | **INTERVENTION** | **Patient life situation,** assessed with the ICAN, is used as a way to identify priorities to address during discharge teaching for the return home, identify domains that are sources of burden or satisfaction/help in patients’ lives and clinical demands. | **Discharge Teaching Guide** to address teaching objectives tailored to **patient activation level,** which is assessed using the **Patient Activation Measure (PAM)** | **Patient Oriented Discharge Summary (PODS)** is a one-page summary with key information, such as the reason for hospitalization or warning signs to be monitored. It will be completed by the patient with the assistance of the discharging nurse. |  |  |
|  | **IMPLEMENTATION** | **Nurses training on teaching intervention components and teaching skills:** prioritization, burden of care, life situation at home, motivational interviewing  Develop and distribute **educational material** | **Nurses training on teaching intervention components and teaching skills**: patient activation concept, adult learning principles  Develop and distribute **educational material** | **Nurses training on teaching intervention components and teaching skills**: PODS  Develop and distribute **educational material** | **Nurses training on teaching intervention components and teaching skills**: motivational interviewing, teach-back, adult learning principles  Develop and distribute **educational material** | **Nurses training on teaching intervention components and teaching skills:** teaching as part of the discharge preparation, involvement of caregivers  Develop and distribute **educational material** |
| Outcomes | **INTERVENTION** | Readiness for hospital discharge  Patients’ experiences with the hospital discharge process | Patient activation  Health confidence  Readiness for hospital discharge  Patients’ experiences with the hospital discharge process  Readmission with 7 days post-discharge | Patients’ experiences with the hospital discharge process  Readmission with 7 days post-discharge  Readiness for hospital discharge | Health confidence  Readiness for hospital discharge  Readmission with 7 days post-discharge | Patients’ experiences with the hospital discharge process |
|  | **IMPLEMENTATION** |  | Nurses attitudes regarding patient activation |  | Behavioural determinants regarding implementation  Nurses attitudes regarding patient activation  Acceptability/Appropriateness/Feasibility Fidelity | Behavioural determinants regarding implementation  Nurses attitudes regarding patient activation  Acceptability/Appropriateness/Feasibility  Fidelity |
| Outcome  Measures | **INTERVENTION** | Readiness for Hospital Discharge Scale (RHDS)  Discharge Care Experiences Survey (DICARES)  Process measures | Patient Activation Measure (PAM) Health Confidence Score (HCS)  Readiness for Hospital Discharge Scale (RHDS)  Discharge Care Experiences Survey (DICARES)  Process measures | Discharge Care Experiences Survey (DICARES)  Readiness for Hospital Discharge Scale (RHDS)  Process measures | Health Confidence Score (HCS)  Readiness for Hospital Discharge Scale (RHDS)  Process measures | Discharge Care Experiences Survey (DICARES)  Process measures |
|  | **IMPLEMENTATION** |  | Clinician support for patient activation survey (CS-PAM) |  | The Determinants of Implementation Behavior Questionnaire (DIBQ)  Clinician support for patient activation survey (CS-PAM)  Discharge Teaching Guide (Fidelity)  Acceptability of Intervention Measure (AIM), Intervention Appropriateness Measure (IAM), and Feasibility of Intervention Measure (FIM) | The Determinants of Implementation Behavior Questionnaire (DIBQ)  Clinician support for patient activation survey (CS-PAM)  Discharge Teaching Guide (Fidelity)  Acceptability of Intervention Measure (AIM), Intervention Appropriateness Measure (IAM), and Feasibility of Intervention Measure (FIM) |
