# Supplementary material for: Implementation and preliminary testing of a theory-guided nursing discharge teaching intervention for adult inpatients aged 50 and over with multimorbidity: a pragmatic feasibility study protocol
Source: Pilot Feasibility Stud. 2021 Mar 17;7:71. doi: 10.1186/s40814-021-00812-4 (PMC7968193; doi:10.1186/s40814-021-00812-4)
Supplement: Supplementary file 5 — Additional file 5. Conceptual basis for the intervention. Link between the programme theory resulting from the realist review, related concepts and theories and intervention components. [file 40814_2021_812_MOESM5_ESM.docx]

Additional file 5. Conceptual basis for the intervention

**CMO7** (Priority concerns about going home) guides the first step in the intervention, which consists of identifying with patients the priorities or concerns regarding their return home. The theoretical underpinning of prioritization in multimorbidity management is the minimally disruptive medicine model (MDM) (17, 18). The MDM sheds light on the often precarious balance between the burden of disease management and/or treatment and older patient’s ability to cope with it (19). Hospitalization usually adds additional treatment to older patients already struggling with the management of multiple chronic diseases in addition to the demands of daily life. With advancing age, these patients have diminished resources in a number of areas, impacting their ability to cope with the additional burden of treatment. Prioritization for the return home is operationalized in the intervention by the Instrument for Patient Capacity Assessment (ICAN), which is the first tool developed to support practice of the minimally disruptive medicine model (52).

**CMO3** (Patient activation) is the core of the intervention because it defines the tailoring of the teaching according to patient's activation level. Patient activation refers to patient knowledge, skills, and confidence for self-management (21). The concept of activation was used as theoretical foundation and serves as the basis for determining what a realistic goal would be for a patient depending on the activation level (49). Patient activation may be disrupted in case of stress or crisis, but is also modifiable and improved by tailored interventions (65). Research shows that patient activation is a factor contributing to self-management and that it is associated with better adherence, health outcomes, lower rates of costly use (hospitalisation, emergency department visit) and lower rate of post-discharge 30-day hospital utilisation (85, 86). The Patient Activation Measure (PAM) has been developed to assess activation and has been used in several studies as a descriptive characteristic of patients during hospitalization or in community healthcare settings to tailor intervention and assess changes across prolonged time frames (21, 49, 56, 87, 88). **CMO1** (Relevancy of teaching content) and **CMO2** (Tailoring teaching delivery) complement CMO7 when the content and the delivery of the teaching is tailored to patients’ activation level and therefore is more relevant to them. This is particularly important for older patients, whose normal physiological changes may impede learning.

**CMO8** (Making sense of the hospital stay experience) is an integral part of completing the patient-oriented discharge summary (PODS). Six health self-management areas are included in this document and correspond to that in the Discharge Teaching Guide used by nurses. Patients complete the PODS during the hospital stay with nurses, who check before discharge that the information contained therein are correct.

All of these CMOs will also be embedded in the training of nurses on how to provide the discharge teaching intervention. **CMO4** (Interviewing skills), **CMO5** (Teaching skills), **CMO9** (Discharge teaching is a care in itself) and **CMO6** (Teachable opportunities) provide specific guidance to clinical nurses on teaching skills and on teaching as part of the discharge preparation. **CMO10** (Involving the caregivers) stresses the importance of including caregivers in the teaching, which will also be taught to the nurses during training. Teaching skills embedded in these CMOs are inventoried in the Theoretical framework to guide patient/family teaching (31). Operationalization of nurses teaching skills described in these CMOs is part of the implementation strategy.
