# Supplementary material for: Implementation and preliminary testing of a theory-guided nursing discharge teaching intervention for adult inpatients aged 50 and over with multimorbidity: a pragmatic feasibility study protocol
Source: Pilot Feasibility Stud. 2021 Mar 17;7:71. doi: 10.1186/s40814-021-00812-4 (PMC7968193; doi:10.1186/s40814-021-00812-4)
Supplement: Supplementary file 6 — Additional file 6. ICAN domains. Life and clinical domains of the ICAN tool. Patients have to classify these as source of burden or satisfaction/help. [file 40814_2021_812_MOESM6_ESM.docx]

Additional file 6. ICAN domains

| Life domains | Care domains |
| --- | --- |
| Family and friends | Take medications |
| Work or finances | Monitor symptoms |
| Free time, relaxation, fun | Manage diet and exercice |
| Spirituality or life purpose | Get enough sleep |
| Living place | Come in for appointment and labs |
| Getting out and transportation | Reduce alcohol use, smoking, etc. |
| Being active | Insurance or support services |
| Social media, TV, or screen watching | Manage stress |
| Emotional life |  |
| Memory or attention |  |
| Food |  |
