# Supplementary material for: Implementation and preliminary testing of a theory-guided nursing discharge teaching intervention for adult inpatients aged 50 and over with multimorbidity: a pragmatic feasibility study protocol
Source: Pilot Feasibility Stud. 2021 Mar 17;7:71. doi: 10.1186/s40814-021-00812-4 (PMC7968193; doi:10.1186/s40814-021-00812-4)
Supplement: Supplementary file 7 — Additional file 7. «Stop » and « Go » progression criteria. Progression criteria that will inform the decision to conduct a larger and definitive trial. [file 40814_2021_812_MOESM7_ESM.docx]

Additional file 7. « Stop » and « Go » progression criteria

|  | Recruitment | Loss to follow-up | Outcome acceptability | Intervention feasibility | Intervention acceptability | Intervention appropriateness | Minimum important difference |
| --- | --- | --- | --- | --- | --- | --- | --- |
| Go | 90 participants (50% of total target recruitment in 4 months)  Consent rate of ≥ 70% | ≤ 20% | ≥ 70% of outcome measure are completed | ≥ 70% of nurses who delivered the intervention scored the feasibility at 16 out of 20 and above. | ≥ 70% of nurses who delivered the intervention scored the acceptability at 16 out of 20 and above. | ≥ 70% of nurses who delivered the intervention scored the appropriateness at 16 out of 20 and above. | ≥ 70% of the participants in the intervention group remained stable or increased their level of activation between measures over time  ≥ 70% of the participants in the intervention group had a RDHS score of ≥ 7 |
| Adapt/change the study process or the intervention | 45-90 participants (25-50% of total target recruitment in 4 months)  Consent rate of between 50 and 70% | 20-40% | ≥ 40% ≤ 70% of outcome measure are completed | ≥ 30% of nurses who delivered the intervention scored the feasibility at 16 out of 20 and above. | ≥ 30% of nurses who delivered the intervention scored the feasibility at 16 out of 20 and above. | ≥ 30% of nurses who delivered the intervention scored the feasibility at 16 out of 20 and above. | ≥ 40% < 70% of the participants in the intervention group remained stable or decrease their level of activation between measures over time  ≥ 40% ≤ 70% of the participants in the intervention group had a RDHS score of ≥ 7 |
| Stop | < 25% of total target recruitment in 4 months  Consent rate of < 50% | > 40% | < 40% of outcome measure are completed | < 30% of nurses who delivered the intervention scored the feasibility at 16 out of 20 and above. | < 30% of nurses who delivered the intervention scored the acceptability at 16 out of 20 and above. | < 30% of nurses who delivered the intervention scored the appropriateness at 16 out of 20 and above. | ≥ 70% of the participants in the intervention group remained stable or decreased their level of activation between measures over time  <40% of the participants in the intervention group had a RDHS score of ≥ 7 |
